# An Autonomous Connectivity Restoration Algorithm Based on Finite State Machine for Wireless Sensor-Actor Networks

**DOI:** 10.3390/s18010153

**Published:** 2018-01-08

**Authors:** Ying Zhang, Jun Wang, Guan Hao

**Affiliations:** 1 College of Information Engineering, Shanghai Maritime University, Shanghai 201306, China; shmtuegh@126.com; 2 Department of Electrical and Computer Engineering, University of Central Florida, Orlando, FL 32816, USA

**Keywords:** wireless sensor-actor networks, network connectivity restoration algorithm, connected dominating set, relocation

## Abstract

With the development of autonomous unmanned intelligent systems, such as the unmanned boats, unmanned planes and autonomous underwater vehicles, studies on Wireless Sensor-Actor Networks (WSANs) have attracted more attention. Network connectivity algorithms play an important role in data exchange, collaborative detection and information fusion. Due to the harsh application environment, abnormal nodes often appear, and the network connectivity will be prone to be lost. Network self-healing mechanisms have become critical for these systems. In order to decrease the movement overhead of the sensor-actor nodes, an autonomous connectivity restoration algorithm based on finite state machine is proposed. The idea is to identify whether a node is a critical node by using a finite state machine, and update the connected dominating set in a timely way. If an abnormal node is a critical node, the nearest non-critical node will be relocated to replace the abnormal node. In the case of multiple node abnormality, a regional network restoration algorithm is introduced. It is designed to reduce the overhead of node movements while restoration happens. Simulation results indicate the proposed algorithm has better performance on the total moving distance and the number of total relocated nodes compared with some other representative restoration algorithms.

## 1. Introduction

The Internet of Things is called the third technology wave in the field of information industry following the Computer and the Internet. As one of the core technologies of the Internet of Things, wireless sensor-actor networks (WSANs) have attracted many researchers’ attention in recent years. WSANs consist of many wireless nodes with perception sensors and reaction actors, which have the mobile functionality to response and feedback according to the change of the surrounding situations. These network nodes can detect environmental information and process the collected information locally and transmit it to a remote base station by multiple hops. WSANs have the feature of autonomously reacting according to the surrounding network state. Usually, WSANs can be used to perform complex tasks such as border protection, reconnaissance exploration in battlefields, effective search and rescue in the ocean, monitor critical areas and track some certain events collaboratively [1,2,3,4,5].

As we know, a WSAN node is usually constrained in its energy and information processing capability, but with the help of more sensor-actors dispersed in the monitoring area, it can achieve a better coverage result and increase the fidelity of the environmental information perception. Upon deployment, nodes are expected to remain reachable to each other and form a connected network in all circumstances [6]. WSANs are sometimes deployed to perform some synergetic tasks in a hostile or unattended environment, and some of the nodes are prone to abnormality or falling out of contact with other nodes, and that will cause them to lose their normal connected state to maintain the network. How to improve the robustness and restore the connectivity between sensor-actors in the event of node abnormity is very important for the effectiveness of WSANs [7,8,9,10], and how to restore the WSANs’ connectivity economically is also of great significance [11,12,13]. 

Aiming at decreasing the movement overhead, a distributed autonomous connectivity restoration method based on finite state machine (DCRMF) is proposed in this work. If there a disconnectedness is detected in one region, according to the criticality of the nodes in this region, DCRMF will determine how to relocate the related nodes and carry out the restoration process autonomously. Moreover, in order to adapt to the dynamic characteristics of WSANs, a critical nodes updating mechanism will be launched after every restoration process is implemented. 

This paper is organized as follows: in Section 2, the related work of the existing connectivity restoration algorithms are introduced. The system model and details of the algorithms are presented in Section 3. In Section 4 and Section 5, the simulation experiment and result analysis are illustrated. Section 6 concludes the paper with a summary. 

## 2. Related Works

The existing connectivity restoration algorithms and fault-tolerant mechanisms of WSANs are divided into two categories: the provisioned and the reactive mechanisms. The provisioned mechanism involves pre-configuring some backup nodes during the network deployment and normal operation. The reactive mechanism performs the real-time restoration through the relocation of the normal nodes [14]. 

The provisioned mechanism is designed to protect the network topology from destruction, and eliminate the possibility of network partitioning. This mechanism usually deploys a redundant node for each key node in the initial deployment phase [15]. When the key node is out of operation, the backup node will be relocated to replace the abnormal node and take on its functions. The node recovery through active spare designation (NORAS) algorithm proposed in [16] belongs to this kind of method. When choosing the appropriate backup node, NORAS only focuses on the significance of the node to the network connectivity and its influence on the degree of coverage of the network. The idea is to find the spare nodes inside the network prior to the abnormity occurring on some nodes, which can then replace the abnormal nodes in case of disconnectedness of the network. 

Because of the large number of redundant nodes in the network with the provisioned mechanism, the network size increases, although the network connectivity is guaranteed. On the other hand the reactive mechanism will not perform the restoration policy until one of the nodes appears abnormal. This method needs to relocate the existing movable nodes in the network to an appropriate location to achieve the connectivity restoration, and this policy is much more suitable for dynamic networks. The Distributed Actor Recovery Algorithm (DARA) proposed in [17] belongs to the reactive mechanism class. The DARA method firstly chooses the appropriate node among the two-hop neighboring nodes of the abnormal node and has that node relocated to the location of the abnormal node. If the relocated node causes a network partition, the restoration algorithm will be executed again until the connectivity of the entire network is restored. This algorithm judges the criticality of the node according to the information of the two-hop neighbor nodes, and restores the connectivity recursively. It is designed to use the local information to restore the connectivity and minimize the moving overhead. In [18], the authors proposed a selection strategy for actors’ substitutes in DARA, in which the substitute selection is based on the nature of the links with neighboring nodes rather than on the degree of the nodes. DARA guarantees that the network connectivity can be restored to the pre-fault level. However, DARA does not rely on the connected dominating set, and also does not fully distinguish the critical nodes [19]. Thus, when the abnormal node does not cause the network partition, the implementation of this algorithm will bring a lot of unnecessary overhead to the network operation. 

In [20], the authors proposed the Least-Movement Topology Repair algorithm (Le-MoToR). This method relies on the local information to attempt to minimize the movement distance of each relocated node and strives to relocate the least number of nodes during the restoration process. Le-MoToR does not impose the nodes’ deployment or store the topology of the network before the node abnormality occurs. That means it cannot utilize the existing path discovery activities in the network to know the structure of the network topology. Because this method does not rely on the overall network topology, the restoration process will not cause more communication overhead. However, in dynamic networks, the distances between the nodes can change at any time, so the performance of this algorithm will be impaired. 

Like the DARA algorithm, the Recovery through Inward Motion (RIM) algorithm also belongs to the reactive mechanism class [21], but RIM only needs to maintain a one-hop information table, and it avoids any complex mechanism to select the alternative nodes. RIM algorithm only involves the movement of one-hop neighbor nodes, and the maximum moving distance for one node does not exceed half of the communication radius of the node, which has the advantage of maintaining the energy balance of the whole network. The RIM algorithm strives to minimize the communication and movement overhead of one moving node. However, the cascaded inward motion results in increases of the total number and moving distances of the relocated nodes, and it also will reduce the coverage ability of the network [22]. 

In [23], the authors proposed the Least Distance Movement Recovery (LDMR) algorithm, which is a distributed approach that exploits non cut-vertex actors in the recovery process. It distinguishes the critical nodes, and the idea is to have a set of direct neighbors of the abnormal node move toward the position of the abnormal node until the abnormal node’s original position is replaced by the nearest non cut-vertex actor. This ensures that each node moves the smallest distance until a new connected domain is formed. If a critical node exists in the neighbor nodes, the algorithm will be executed again. Although the algorithm distinguishes the critical nodes from the non-critical nodes, the algorithm only cares about the moving distance of each single node and it does not care about the total moving distances of all the relocated nodes. 

In this paper, we propose the algorithm of distributed autonomous connectivity restoration method based on finite state machine (DCRMF). The primary goal is to reduce the total movement overhead during the restoration process. The mechanism of finite state machine is performed to increase the adaptability of the algorithm in dynamic networks. The node state is updated at any time according to the states of the surrounding nodes. The simulation results indicate that the proposed algorithm has better performance on the total moving distances and the number of total relocated nodes compared with other connectivity restoration algorithms.

## 3. System Model and the Algorithm Details

### 3.1. Network Model and Some Definitions

We assume that the system is a network with randomly dispersed wireless sensor-actor nodes. Like in [24], each node obtains the information of its location through its own hardware devices periodically, broadcasts its state and exchanges information with its neighbor nodes to maintain the 1-hop and 2-hop neighbor tables. Each node in the network can move autonomously. Here we do not consider the communication influences of MAC layer of the network.

The initial connected sensor network with *M* movable sensor-actors is represented by an undirected graph *G* = (*V*, *E*), where *V* = (1, …, *M*) denotes the set of vertices indexed by the set of mobile sensor-actors *i* ∈ [1, …, *M*], and the edges set *E* = {*e_ij_*, (*i*,*j*) ∈ *V*} denotes the unordered pairs which specifies the bidirectional communication links existed between the respective sensor-actors. The Euclidean distance between sensor-actors *i* and *j* is represented by $d_{ij}=\|\xi_{1}-\xi_{j}\|$, where $\xi_{i}$ represents the coordinate of sensor-actor *i*. The edge *e_ij_* ∈ *E* exists if and only if *d_ij_* ≤ *R*, where *R* is the communication range of the sensor-actors. The network has homogenous configuration for all sensor-actors. Every sensor-actor in the network is equipped with the same computing functions and has the same communication range. According to the network model above, we can make the following definitions:

**Definition** **1** (connectivity)**.** An undirected graph G is connected if and only if there is at least one path for any two vertices.

**Definition** **2** (1-hop neighbors)**.** *Define j as the 1-hop neighbor of i, i.e., j* ∈ *N_i_, if and only if e_ij_ ≤ E, where N_i_ is the 1-hop neighboring set of i.*

**Definition** **3** (2-hop neighbors)**.** *Define k as the 2-hop neighbor of i, i.e.,* $k\in N_{i}^{2}$*, where* $N_{i}^{2}$ *is the 2-hop neighboring set of i, if and only if there exists at least one route between i and k in G with two hops.*

**Definition** **4** (node degree)**.** If there are k nodes in N_i_, define k as the node degree of node i, i.e., D_i_ = k, it means the number of node i’s neighbors is k.

**Figure 1:**
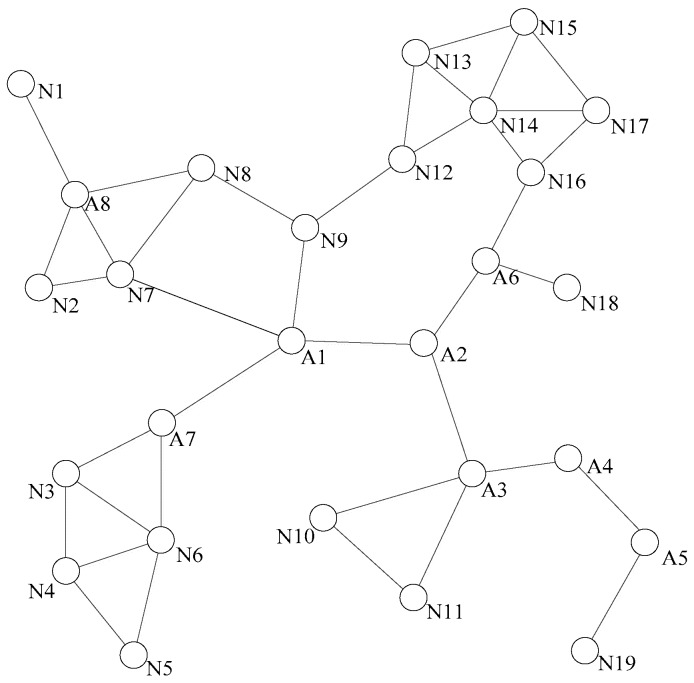
The topological diagram of a normal sensor-actor network.

Figure 1 is a schematic diagram of topology relationships between sensor-actor nodes in a network. This network is connected, that means that there is at least one path between any two nodes. Each node gets the states of the surrounding nodes through the heartbeat information detection. 

When an abnormity occurs on a leaf node, the other nodes are not affected. However, if the abnormal node is a gateway node, connectivity of the network will be severely destroyed, and such a node is called a cut-vertex [25]. The abnormity of the cut-vertex will make the network partition and a number of nodes cannot find the communication link. As a result, the normal operation of the network applications will be seriously affected.

As shown in Figure 1, the abnormity of node N1 does not affect any other node in the network. However, if node A7 falls into abnormity, nodes N3, N4, N5, and N6 cannot normally communicate with other nodes in the network because there is no communication link between them.

In view of the critical role of the cut-vertex in network connectivity restoration, this paper focuses on how to improve the efficiency of network connectivity restoration by detecting the cut-vertexes and managing nodes’ relocations for the purpose of reducing the total travelled distances and the total number of relocated nodes.

The main objectives of the proposed algorithm are as follows: (i) restore the connectivity of the abnormal network real-timely by using the distributed autonomous restoration algorithms. (ii) Meanwhile, optimize the energy consumption during the implementation procedure. 

The specific measures of the proposed algorithm is to keep the minimum number of the dominating nodes and move the nearest non-dominating node to replace the abnormal node. The steps are as follows:

(1) For the initial topology, calculate the connected dominating set and optimize the set with finite state machines mechanism.
(2) When an abnormal node is found in the network, select the nearest non-dominating node in the neighbor node set as the optimal alternative node according to these specific conditions: distance, node degree, and the criticality.
(3) Replace the abnormal node, and update the state of the connected dominating set dynamically.

### 3.2. Connected Dominating Set Generation and Cut-Vertex Detection

Due to the existence of redundant nodes in the connected dominating set when using the traditional resolving algorithms [26], the increase of the number of nodes in the connected dominating set will increase the possibility of unnecessary nodes’ moving for restoration, and this will lead to additional unnecessary moving overhead during the restoration procedure. 

In addition, the movements of the nodes in the network will cause a change of network topology, the dominating relationships of the nodes will also be changed accordingly, and this is not applicable to the traditional connected dominating set forming. Therefore, we introduce the finite state machine model to reduce the redundant number of the cut-vertexes by filtering the members of the connected dominating set. This mechanism makes the connected dominating set updated in each round, and has the system adapt to the dynamic changes of the surrounding topological relations. 

Before we discuss the method, some notions must be clarified. The cut-vertex is the critical node which connects two or more partial networks. Dominating nodes are the nodes which constitute the skeleton of a connected network. Obviously, the non-dominating nodes are the nodes whose abnormity cannot cause the network partitioned. 

The simplest method to test whether an abnormal node is a cut-vertex is to use the Depth-First Search algorithm to conduct a flooding, but this requires a very large communication overhead. In order to reduce the communication overhead brought by the detection of cut-vertexes, the concept of connected dominating set is introduced. As long as the nodes in the connected dominating set are all in the normal state, each node in the network can communicate with each other. It is possible to reduce the possibility of initiating the connectivity restoration procedure by reducing the number of the nodes in connected dominating set, and this measure can certainly avoid the unnecessary overhead of nodes moving. The determination of the minimum connected dominating set has been proved to be a NP-complete problem. As we know, it is difficult to avoid the redundant nodes in the connected dominating set by the traditional solutions [27]. Therefore, in order to improve the restraining control for the redundant nodes in the connected dominating set, we introduce the finite state machine model in the restoration process to filter the member nodes in the connected dominating set, and update the connected dominating set periodically after each execution of network restoration procedure is completed. This mechanism can ensure the network connectivity restoration procedure maintains the appropriate response to the changes of the nodes’ states. 

The four identities for nodes information in the states transition diagram are as follows: node number, dominant node, color and the state, which can be used to describe the nodes with different states. If the node’s color is marked as black, it means that the node is the dominant node and it joins into the connected dominating set, if a node is marked as gray, it indicates that the node is a non-dominating node, and the white node represents the initial state. The state of a node is represented by one of the four numbers: 0 to 3, the state 0 represents the node is in the initial state, the state 1 represents the node is in an unstable state, the state 2 represents the node is in a non-dominating state, and the state 3 represents the node is in a stable state of dominance.

After the restoration algorithm is executed to deal with a node falling into abnormity, the finite state machine needs to be executed to update the local connected dominating set. The specific procedure is as follows:

Step 1: If there is a neighbor node of node *i* whose state value is 3, the state of node *i* needs to be transferred to 0.
Step 2: If node *i* is in state 0, it sends status information to *N_i_* (*N_i_* is the set of node *i*’s neighbor nodes). For any two nodes *V*,*U* ∈ *N_i_*, if the node *V* ∈ *N_u_*, mark the node *i* as in state 2, otherwise mark the node *i* as in state 1.
Step 3: If node *i* is in state 1 and the states of its neighbor *N_i_* do not include 0, meanwhile, the node satisfies the following conditions: (1) ∃*V* ∈ *N_i_*, the color of node *V* is black, where *N_i_* ⊆ *N_v_* and *D_i_* ≤ *D_v_*. (2) *i* = *Del*(*V*1,*V*2,*i*), where *V*1,*V*2 ∈ *N_i_*, both of the nodes belong to black and they are connected each other. If the node *i* satisfies the above conditions, the node state should be transferred to state 2, otherwise transferred to state 3, where, we define *Del*(*V*1,*V*2,*V*3) as a deletable set, and its value is a collection of the deletable black nodes. It means if we delete these nodes, it can still form a connected dominating set by the remaining nodes, and the network with the rest nodes is still connected. If (*N_V_*_1_ ∩ *N_V_*_2_) ⊇ *N_V_*_3_, then *Del*(*V*1,*V*2,*V*3) = *V*3.
Step 4: When the node *i* is in state 2 and all the neighbor nodes in *N_i_* are not in state 0 or state 1, select the node which has the minimal node number in the *N_i_* as the dominant node and transfer the state of node *i* to state 1.

**Figure 2:**
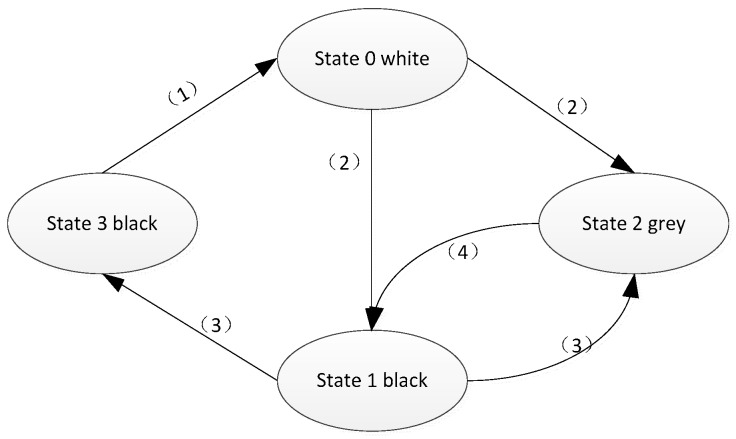
The node states transition diagram.

The states transition mechanism above can be illustrated as Figure 2. The pseudo code of the update algorithm to the connected dominating set is as indicated in the following Algorithm 1, in which the node *i* is an arbitrary abnormal node in the network, and node *j* represents the arbitrary neighbor of the abnormal node *i*.
**Algorithm 1** Update the Connected Dominating Set**1. begin****2. for *j*←*Neighbor_hop*1(*i*) do****3.**    **if *isDenominator*(*j*) = *true* then****4.**    ***states*(*j*)←0****5.**    **end if****6.**    **if *states*(*j*) = 0 & (∀*V*,*U*∈*N_i_*,∃*V*∈*N_u_*) then****7.**    ***states*(*j*)←2****8.**    **else *states*(*j*)←1****9.**    **end if****10. if**
*states*(*j*) = 1 **& *colour*(*Neighbor_hop1*(*j*)) = *black* then****11.**   *states*(*j*)←2**12.**   **else**
*states*(*j*)←3**13.**   **end if****14. if**
*states*(*j*) = 2 **&**
*states*(∀*Neighbor_hop*1(*j*)) > 2 **then****15.**   *states*(*j*)←1**16.**   **end if****17. end for****18. end**

Step 1 is the key stage of the update algorithm to the connected dominating set. When the state values of the abnormal node or its neighbor nodes are changed, it is necessary to run the update algorithm to keep the network state at the latest status. In the case of the dynamic network topology, the criticality of the nodes will always be changed. In order to solve this problem, the finite state machine model is introduced to ensure that the connected dominating set can be updated regularly.

### 3.3. Detailed Implementation of Connectivity Restoration Algorithm

The basic idea of the connectivity restoration algorithm is that it can determine whether the node is a cut-vertex through the generation algorithm of connected dominating set. If the node is abnormal according to the heartbeat information detection between the node and its neighbor nodes, and this node is a cut-vertex in the topology, then the system will select an alternative node from the neighbor nodes and have it move to the location where the abnormal node stays. If the alternative node is still a dominant node, the restoration algorithm will be implemented in cascaded mode until the alternative node is a non-dominating node. The algorithm is divided into two steps: (i) select the best alternative node; (ii) relocate the alternative node to the abnormal node’s location. In the choice of alternative nodes, it strives to decrease the overhead of performing the restoration algorithm according to the principle of minimizing the total moving distances and the total number of the relocated nodes. 

#### 3.3.1. Choose the Optimal Alternative Nodes

The goal of choosing an alternative node is to replace the abnormal node timely and efficiently, and restore the connectivity of the network. Therefore, the choice of the alternative node should be based on the criticality of the node and the distance from the abnormal node to the alternative node. First, what we do for considering the criticality of the node is to judge whether it belongs to the dominating node, which affects the number of the nodes involved in the entire restoration process, and the prior choice of non-dominating nodes can minimize the number of relocation nodes. Second, it needs to consider the distance between the neighbor node and the abnormal node, selecting the node with short distance can guarantee the minimum movement cost of each relocation process. In addition, if a cut-vertex is moved, the number of the effected nodes will quite increase, and more movement costs will be inducted markedly. The detailed selection process is as follows: if the abnormal node *i* is detected by the heartbeat information between the nodes, select the non-dominating node *j* in the one-hop neighbor node set *N_i_* of node *i* with the nearest distance according to its two-hop information table $N_{j}^{2}$ as the alternative node *A_s_*. If there is no non-dominating node in *N_i_*, select the dominating node with the smallest node degree and the nearest distance as the alternative node *A_s_*. In the process of choosing the optimal alternative node, the finally selected node is marked as *s*:

(1) $$Ds=min(d_{i1},d_{i2},d_{i3},\cdots ,d_{in})$$

where *D_s_* denotes the distance between the optimal alternative node and the abnormal node, and this distance is the smallest value of the distances between the abnormal node and all its neighbor nodes, and *d_ij_* can be calculated with the coordinates of these nodes:

(2) $$d_{ij}=\sqrt{{\left(x_{i}-x_{j}\right)}^{2}+{\left(y_{i}-y_{j}\right)}^{2}}\;\forall i=1,2,3,\cdots ,n$$

where *i* represents the node number of the abnormal node, which can be the integer value from 1 to *n*, and *j* represents the node number of the arbitrary neighbor nodes of the abnormal node *i* [28].

**Figure 3:**
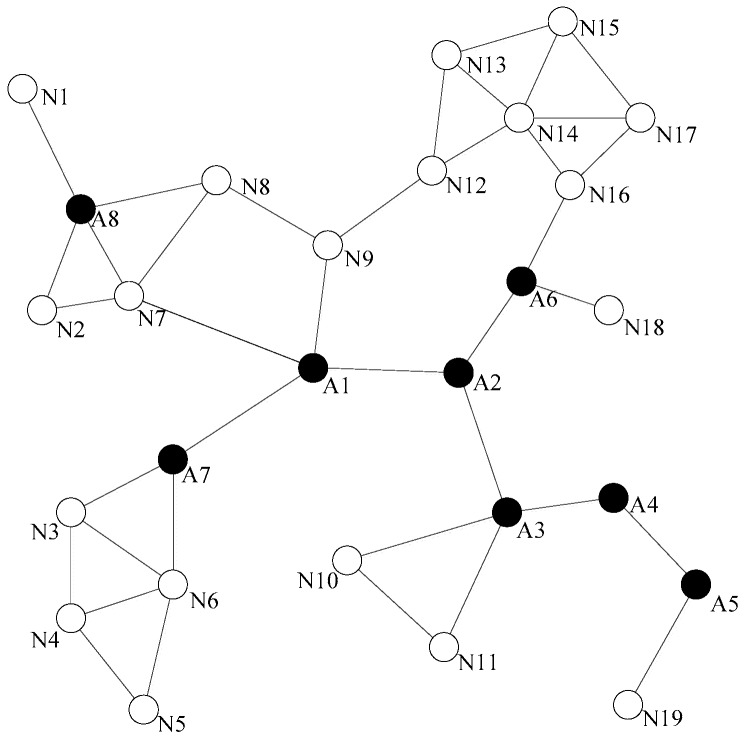
The network topology diagram after the detection of the dominating nodes.

Figure 3 shows a situation of the network topology diagram after the detection of the dominating nodes. When a non-dominating node becomes abnormal, it is not necessary to initiate the network connectivity restoration, because its absence does not pose a threat to the connectivity of the entire network. When the dominating node falls to abnormity, for example, node A1 is abnormal, its neighbor nodes A2, A7, N9, and N7 are aware of its abnormality through the heartbeat information detection, each neighbor node can learn all the neighbor nodes of A1 from the two hops neighbor information, and calculate the optimal alternative node, respectively. A2 and A7 are firstly excluded because they are the dominating nodes. N9 can be chosen as the optimal alternative node from N7 and N9, because N9 is nearer to A1. However, in a sparse network, the connectivity degree of a node is usually 1, and the rule for selecting the alternative node is slightly different from the DARA algorithm. First, considering the node degree of the adjacent nodes, it is more likely that the node with large node degree could be chosen as the alternative node. This is because the bigger the node degree is, the greater the possibility of non-dominating node could exist around. Then, we shall consider the distances from the alternative node to the abnormal node. In Figure 3, if node A4 becomes abnormal, because it does not have any non-dominating nodes around, we can only select the alternative node from among the dominating nodes A3 and A5. Considering that the node degree of A3 is 4 and the node degree of A5 is 2, A3 will be selected as the optimal alternative node. 

#### 3.3.2. Nodes Relocation 

Relocation is defined as an autonomous movement which means a node moves to another position without artificial assistance. The alternative node gets the abnormal node’s location by querying the one-hop information table. The local connectivity is restored by moving the alternative node to the coordinates where the abnormal node remains, and the alternative node will also replace the function of the abnormal node. If the selected alternative node is a non-dominating node, the restoration process will be ended after the relocation, and the broken connectivity by the abnormal node could be rebuilt. As shown in Figure 3, it assumes that node A1 is abnormal, and the abnormity of A1 will interrupt the inter-node communication link between A2 and A7 areas, as well between A2 or A7 area and N7, N9 areas. 

**Figure 4:**
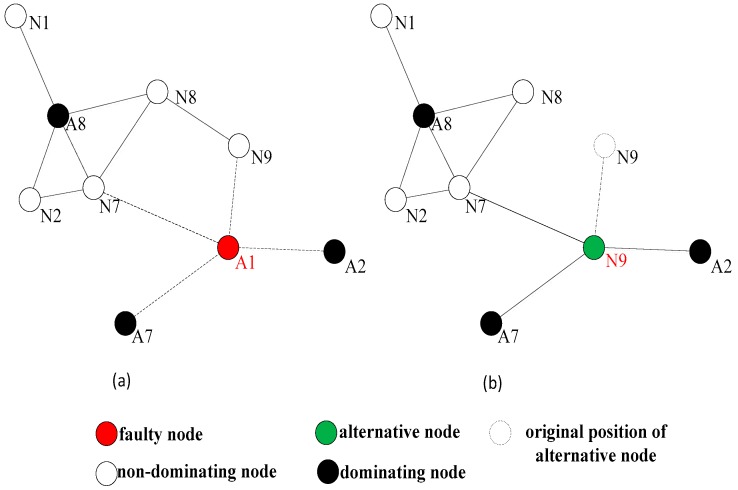
Connectivity restoration process with single node relocation.

**Figure 5:**
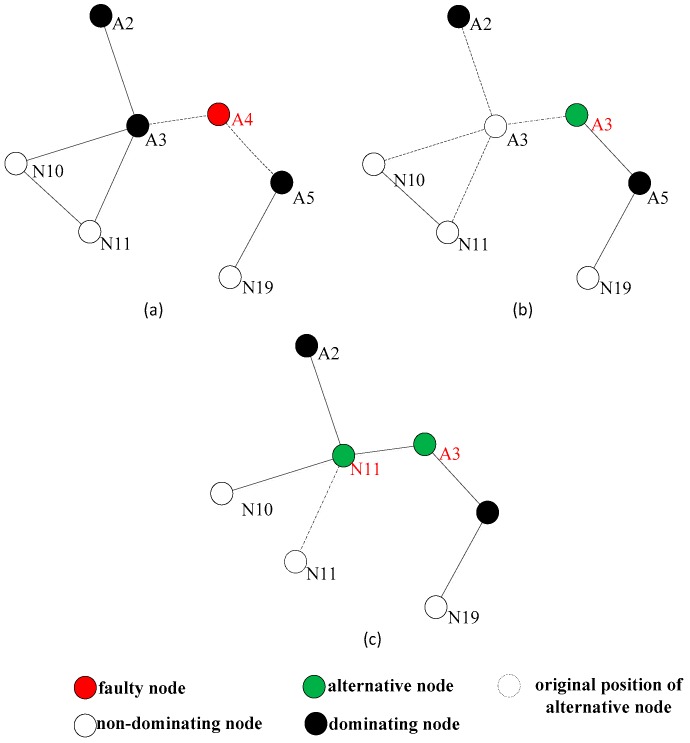
Connectivity restoration process with multiple nodes relocation.

As shown in Figure 4, the optimal alternative node is N9. After N9 moves to the original position of A1, although the 1-hop connection between N8 and N9 will be disconnected, the connectivity between the partitioned networks could be restored, and N8 still can communicate with N9 through N7 as well. However, in the sparse network, the probability of choosing the dominating node as the alternative node will be high, and the relocation of the dominating node recursively causes multiple nodes participate the relocation until the relocated node is a non-dominating node. As shown in Figure 5, the restoration process involves multiple nodes relocation. If A4 falls to abnormity, because its neighbors are all the dominating nodes, A3 will be selected as the alternative node which has the biggest node degree. The movement of A3 still causes the network partition, so it needs to conduct the connectivity restoration again. N11 could be selected to relocate to the original location of A3. Because N11 is a leaf node, which belongs to the non-dominating nodes, the restoration process will be ended here, and the network connectivity is restored. 

#### 3.3.3. The Restoration Algorithm’s Pseudo Code

The pseudo code of the restoration algorithm is shown in Algorithm 2. This restoration algorithm is a distributed algorithm and each node only needs to maintain its two-hop information. The term *i* represents the number of the abnormal node in the network, *j* is the one-hop neighbor node of the abnormal node *i*. The variable *hop* denotes the hop number from the abnormal node to other node. The function *getPosX*(*i*) returns the abscissa of node *i*, and *getPosY*(*i*) returns the ordinate of node *i*, *setPos*(*j*,*destinationX*,*destinationY*) means the action of moving the node *j* to the new coordinate (*destinationX*,*destinationY*) autonomously. Function *CDS_update*(*j*) denotes the execution process of Algorithm 1. Function *SelectNode*(*i*) returns the optimal alternative node selected from the neighbor nodes of node *i*.
**Algorithm 2** Connectivity Restoration**ConnectivityRestore(*i*)****1.**   **begin****2.**   **if**
*isDenominator*(*i*) = *true*
**then****3.**    *j*←*SelectNode*(*i*)**4.**    *destinationX*←*getPosX*(*i*)**5.**    *destinationY*←*getPosY*(*i*)**6.**    *setPos*(*j*,*destinationX*, *destinationY*)**7.**    *CDS_update*(*j*)**8.**   **end if****9.**   **end****SelectNode(*i*)****10.**  **if**
*hop*⇐2 **then****11.**   **for**
*m←Neighbor_hop*1(*i*) **do****12.**    **if**
*isDenominator*(*m*) = *true*
**then****13.**    **add**
*m*
**to**
*CandidateSet***14.**    **end if****15.**   **end for****16.**   **if**
*CandidateSet* = *NULL*
**then****17.**    *hop*←*hop*+1**18.**    **return**
*SelectNode*(*Neighbor_hop*1(*i*))**19.**   **else return** min(*distance*(*i*,*CandidateSet*))**20.**   **end if****21.**  **else****22.**   **return**
*node*←min_*distance*(*Neighbor_hop*1(*i*))**23.**  **end if**

#### 3.3.4. Distributed Regional Restoration Strategy

Multiple node abnormities sometimes appear in WSANs, and the network connectivity will be damaged. In order to deal with multiple node abnormities, a distributed regional restoration strategy is introduced. Here we only consider the situation that the restorations for multiple abnormal nodes in different regions are independent and they are not affected by each other within one-hop distance. If there is intersection in the one-hop neighbors, that means there is an abnormal node in another abnormal node’s neighbor node set, and they stay in the same region. On this occasion, the abnormal node can be masked in the neighbor nodes set, because it provides no help to the network connectivity restoration. After the restoration, this masked abnormal node will be released, and the next step will focus on the restoration for this abnormal node, so with more computational resource consumption, this conflict occasion could be separately resolved as an independent restoration procedure by this “mask” mechanism. 

Therefore, we should define the concept of the “region”. If two nodes are in different regions, that means there is no such intersection among their one-hop neighbors. For two nodes *i* and *j* in the network, if the intersection set of *N_i_* and *N_j_* is empty, the nodes *i* and *j* are in two different regions respectively. The different regions can be mathematically satisfied the condition as ∀*i*,*j* ∈ *V*, *N_i_* ∩ *N_j_* = ∅. Under this condition, the restoration of multiple abnormal nodes could be deal with separately in different regions in the network. The process of conducting this distributed regional restoration strategy is as follows:

(i) Find an abnormal node in one region (we assume that, if after polling five times from a nearby sending node, there is no normal response from the node, this node could be regarded as an abnormal node in this region). When dealing with an abnormal node in one region, the abnormal nodes in other region would not affect this restoration process. Thus, the relative information from the abnormal nodes in other regions could be blocked for this region’s restoration, since that information is no help to this region’s restoration. The restorations in different regions can be performed separately.
(ii) The single node restoration algorithm based on finite state machine is conducted to restore the connectivity for this local region.
(iii) Complete the restoration procedures for all the regions with abnormal nodes.
(iv) The whole network connectivity could be restored after the regional connectivity restoration.

## 4. Simulations

In this paper, the algorithm is designed and performed on the Matlab2010b simulation platform, and it is assumed that the network is composed of movable wireless sensor-actor nodes which are randomly distributed in an area of 800 m × 800 m. Experimental parameters setup involves two kinds of scenarios: (i) variable N of the total number of wireless sensor-actor nodes can be changed in the interval of [80, 200] over the step interval of 20, which can form the different densities’ network topology, meanwhile it keeps the communication radius r as a fixed value in the interval of [80, 200] [29]. (ii) The total number of wireless sensor-actor nodes is a fixed value in the interval of [80, 200], and the communication radius r can be changed in the interval of [80, 200] over the step interval of 20. The communication influences of MAC layer of the network are not considered [30] here. The experimental parameters are set up as Table 1. 

**Figure 6:**
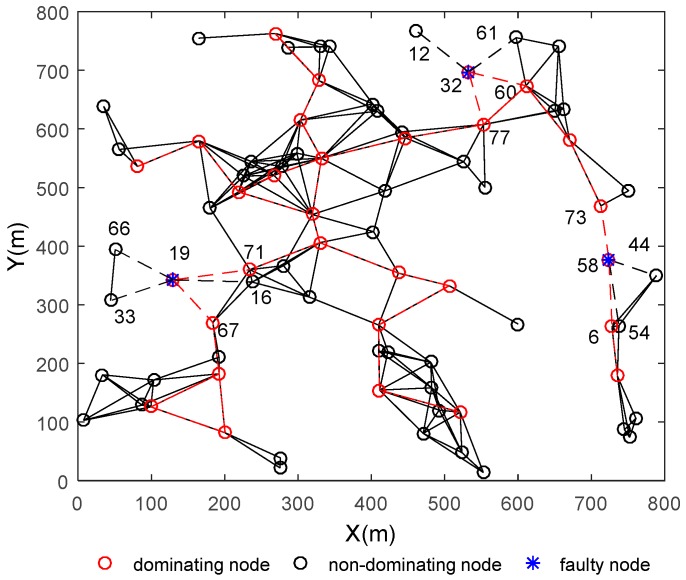
The initial network situation in which the total node number is set as 80 and communication range is set as 120 m.

The regional connectivity restoration strategy for multiple abnormal nodes will be performed according to Section 3. The area of the deployment region is a square of 800 m × 800 m, the total node number is 80, and the communication range is 120 m. There are three abnormal nodes numbered as 19, 32 and 58, respectively, in Figure 6. All the nodes are the WSAN nodes inside. 

This topology is formed by deploying the nodes randomly. The black circles represent the non-dominating nodes and their abnormities cannot affect the connectivity of the whole network. The black lines are the general wireless communication links between the non-dominating nodes, and their disconnection does not cause the partition of the network. The red circles represent the dominating nodes in the connected dominating set which are calculated and updated by the finite state machine model regularly, and the links between these dominating nodes is colored with red so that we can see the backbone of the network clearly. The nodes filled with blue stars represent the abnormal nodes, and dashed lines are used to indicate the missing link caused by node abnormality. This figure only shows one of the topology situations. In fact, the subsequent analysis results are based on the average values obtained from 100 randomly generated different topologies. 

**Figure 7:**
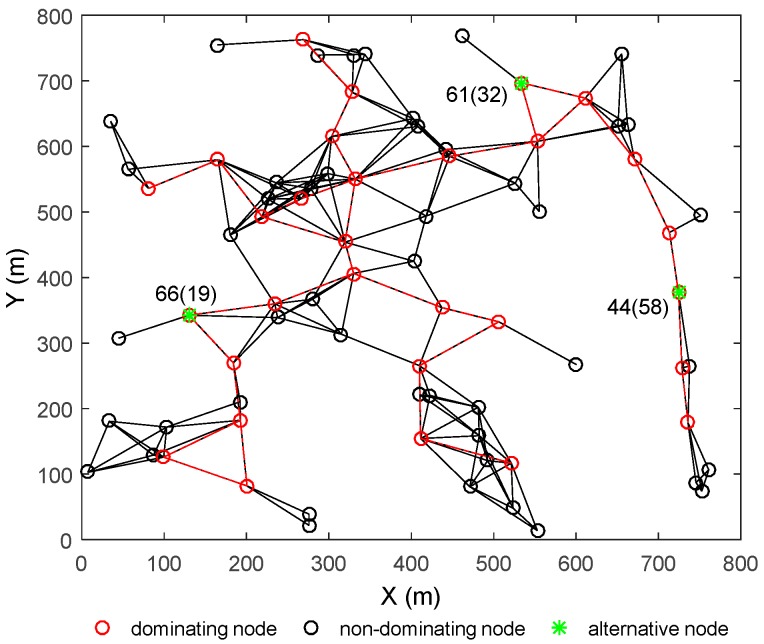
The network topology after restoring the connectivity for the three abnormal nodes.

In the case of multiple node abnormalities, a distributed regional restoration strategy is introduced to restore the network connectivity. The initial network topology is shown as Figure 6. The three nodes filled with blue stars represent the three random abnormal nodes with IDs 19, 32 and 58, respectively. These abnormal nodes will cause the regional network to partition, and the connectivity of the network is destroyed. According to the strategy in Section 3, the proposed algorithm could solve the problems as follows: (i) Judge whether these abnormal nodes belong to separate regions in the network according to the judging criteria in Section 3; (ii) if these abnormal nodes are in different regions, they can be dealt with separately by the single node restoration algorithm based on finite state machine. In Figure 6, the abnormal nodes No. 19, 32 and 58 are in different regions, after conducting the restoration algorithm, the nearest non-dominating node are selected, move to replace these abnormal nodes, and the network state after restoration is shown in Figure 7; (iii) if there is more than one abnormal node in the same region, the “mask” mechanism will be initiated, and some independent restoration procedures applied separately; (iv) the connectivity of the whole network is restored finally. 

Figure 7 shows the network topology after performing the distributed regional restoration strategy for the abnormal nodes. The red circle filled with green stars are the alternative nodes which are from the nearest non-critical neighbors of the abnormal nodes. The original abnormal nodes represented by the red circles filled with blue stars are all replaced by the alternative nodes 66, 61 and 44 respectively, which migrate to the positions where the abnormal nodes stay originally. We just put the original abnormal nodes’ No. in the brackets after the alternative nodes’ No., respectively, their relationship to each other is easy to see. The partitions of the network are eliminated. 

The total number of the nodes and the communication radius of the node in the network can be regarded as the independent variables to analyze and evaluate the performance of the algorithms in different size and sparse states of the network. The impacts of these two variables are explained as follows:

(i). Total Moving Distance

This is the key parameter to evaluate the performance of the algorithm. This parameter is the sum of the moving distance of all the relocated nodes involved in the restoration process [31]. Since the movement consumes a large amount of a node’s energy, the most effective way for saving the energy of the network is to reduce the total moving distances of all the nodes. It is an important metric to evaluate the performance of the network connectivity restoration algorithms.

(ii). Number of Relocated Nodes

This parameter represents the total number of the sensor-actor nodes participating in the relocation movement during the restoration process [32]. The smaller the number of the nodes involved in the movement is, the slighter the impact on the topology of the whole network will be. The smaller the number of relocated nodes is, the higher the efficiency of the algorithm will have.

DARA, RIM, LDMR and the proposed DCRMF algorithms are all the reactive connectivity restoration algorithms, thus we use that three representative algorithms as the comparison objects in the subsequent experiments. Table 2 is the comparison of network characteristic parameters for the four reactive algorithms. In this table, r denotes the communication range, N denotes the total number of the nodes, and N_d_ denotes the total number of the dominating nodes. The table indicates that, compared with the other three algorithms, the proposed DCRMF algorithm has obvious advantages on the theoretical value of the total moving distance and the number of relocated nodes. Although RIM algorithm has obvious advantages on the moving distance of a single node and the requirements of the topology information, the number of total relocated nodes is very large, and this will lead to more increase of the total movement overhead of the network. 

## 5. Results and Analysis

In these experiments, the algorithms will be evaluated in 100 different topologies generated randomly, and the analysis results are the average values based on these different network topologies accordingly. As shown in Table 2, DARA, RIM and LDMR algorithms are selected as the contrastive algorithms compared with the proposed DCRMF algorithm.

### 5.1. The Effect of Total Number of Nodes on the Performance of the Algorithms

**Figure 8:**
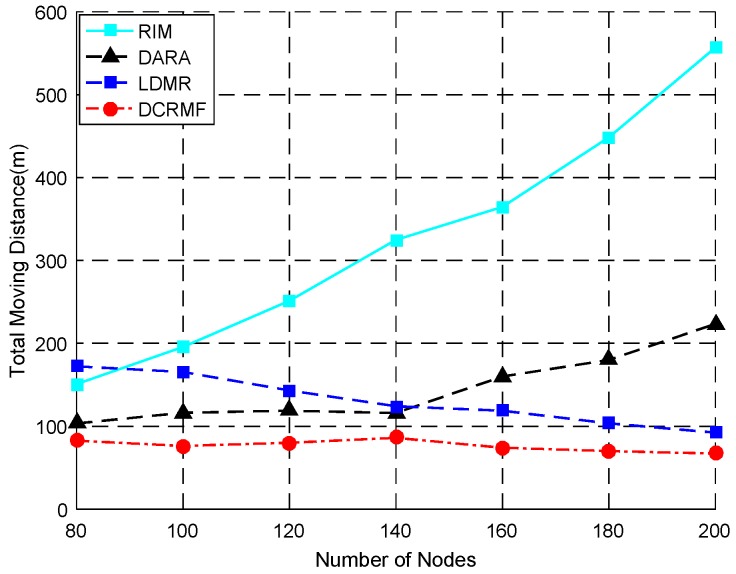
The relationship between the total moving distance and the total number of the nodes in the network by using these four algorithms.

The relationship between the total moving distance and the total number of the nodes is shown as Figure 8. The detailed contrast data of different algorithms for total moving distance is shown in Table 3. N denotes the number of nodes in the network. TMD denotes the total moving distances. The total moving distance during the restoration changes with the varying of the number of the nodes of the network. Table 3 illustrates that the total moving distance of DCRMF is less than the other three algorithms, and as the number of nodes increases, the total moving distances of DCRMF and LDMR slowly decrease as well, but DARA and RIM are with the opposite cases. 

When the RIM restoration algorithm is applied to large-scale networks, once a node becomes abnormal, it will cause a large number of nodes to move, while the use of the DARA restoration algorithm does not involve a large number of nodes, however, because there is no distinction between dominating nodes and non-dominating nodes in this algorithm, the relocation activity will be easier to conduct in cascade. In most cases, the proposed DCRMF algorithm only relocates the non-dominating nodes from the neighbor nodes, and the total moving distance will be reduced accordingly. In some networks, the increase of the total number of the nodes will lead to the increase of the density of the network, thus it is easier to find a closer alternative node. 

As seen in Figure 8, as the total number of nodes increases, the total moving distance of RIM increases exponentially, and the curve of DARA is also on a rising trend, but the curve trend of the proposed DCRMF declines a little, and it has an advantage in the total moving distance as a whole. Although the tendency of LDMR is also declining, its total moving distance value is bigger than that of DCRMF in all the situations. 

**Figure 9:**
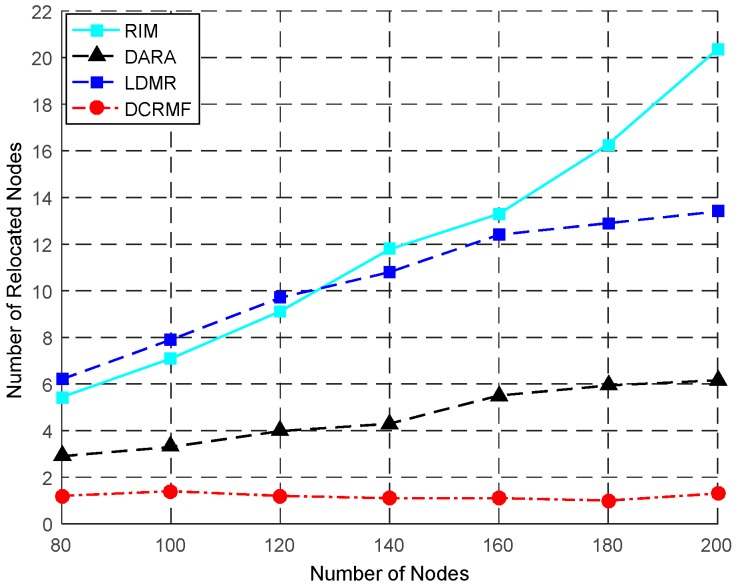
The relationship between the number of relocated nodes and the number of nodes by using these four algorithms.

The relationship between the number of the relocated nodes and the total number of the nodes is shown in Figure 9, and the detailed contrast data is shown in Table 4. In the relocation process, LDMR involves each neighbor node in order to achieve a minimal value on the moving distance of each node, so the total number of relocated nodes is very large. As for the proposed DCRMF, it distinguishes between the dominating nodes and non-dominating nodes, and node degree will be considered only in the case where no dominating nodes are around, so the probability of the cascaded motion occurring during restoration will be greatly reduced. In most cases, the average number of the relocated nodes is about 1 when the DCRMF is applied. The proposed algorithm has an obvious advantage in the number of relocated nodes compared with the other three algorithms. 

In Table 4, N denotes the number of nodes of the network. NRN denotes the number of the relocated nodes. The average number of relocated nodes got from 100 random topologies changes with the number of nodes varying. Obviously, Table 4 shows that the average number of the relocated nodes in DCRMF is less than with the other three algorithms. The greater the size of the network is, the more obvious the advantage DCRMF will have. 

**Figure 10:**
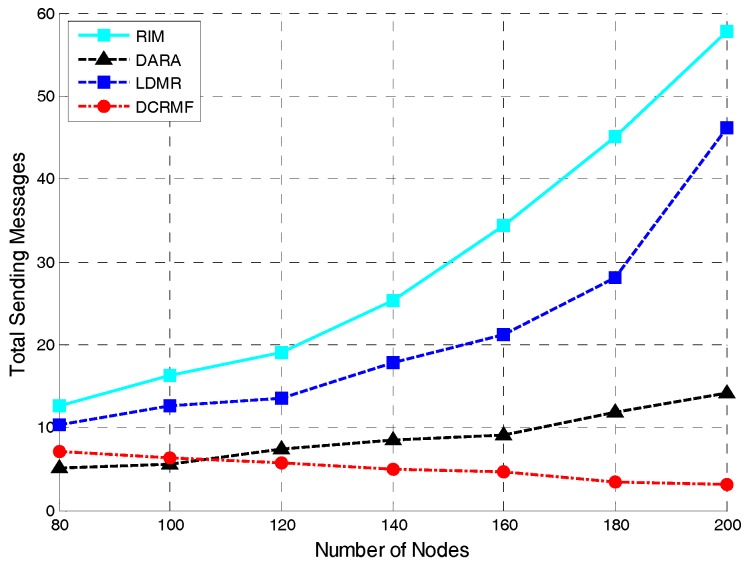
The relationship between the total sending messages and the number of nodes in the network by using these four algorithms.

The relationship between the number of nodes and the total messages sent is illustrated as Figure 10. The proposed DCRMF algorithm still has an advantage on the total messages sent. As seen in Figure 10, in order to complete the restoration process, the total number of messages sent with the of RIM algorithm increases exponentially as the number of nodes increases. In the case of a small number of nodes in the network, the number of messages sent by the DARA algorithm is less than that in DCRMF algorithm, but in a network with a large number of nodes, the DCRMF algorithm has more advantages. This is mainly because, as the number of nodes increases, the probability of finding non-dominating nodes around the abnormal node will increase, and the number of nodes which need to send messages during the restoration process decreases. Table 5 shows how the detailed average number of total sent messages for different algorithms changes with the varying number of nodes. N denotes the number of nodes of the network, and TSM denotes the total sent messages in the restoration process.

### 5.2. The Effect of Node Communication Range on the Performance of the Algorithms

The change of node communication range will affect the density of the network formation. The analysis of the influence of communication range variation on the algorithms also needs to be discussed for different network distributions. 

**Figure 11:**
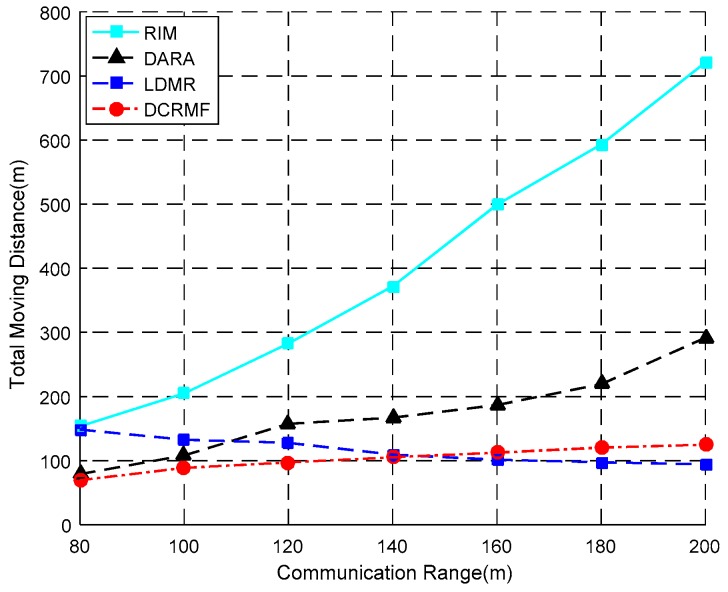
The relationship between the total moving distance and the communication range by using the four algorithms.

The relationship between the total moving distance and the communication range is shown in Figure 11, and some detailed contrast data is shown in Table 6. TMD still denotes the total moving distances, and R denotes the communication range of the nodes in the network. With the same radius and at a lower node density, DARA and the proposed DCRMF algorithms have almost similar performance. This is because the connectivity of the network with lower nodes density in most cases is the 1-connectivity networks. In this case, the nodes except the leaf nodes are all the dominating nodes. 

In Figure 11, LDMR has better performance when the communication range becomes large. The reason is that the maximum distance that each node can travel is only the half of its communication range in this experiment. As the communication range is increasing, the number of relocated nodes will decrease as well, and this advantage will be gradually reflected.

The implementation results of DCRMF and DARA are very similar. However, in high density networks, there are more non-dominating nodes, the alternative node has large selection space, and the superiority of DCRMF can be better reflected. When the communication range is greater than 100 m, DCRMF can save 30–50% of the movement overhead compared to DARA.

In Table 6, the number of relocated nodes varies with the varying communication range. The table illustrates how the total moving distance in DCRMF is less than those in other three algorithms, and as the communication range increases, the total moving distance in DCRMF slowly increases as well. 

**Figure 12:**
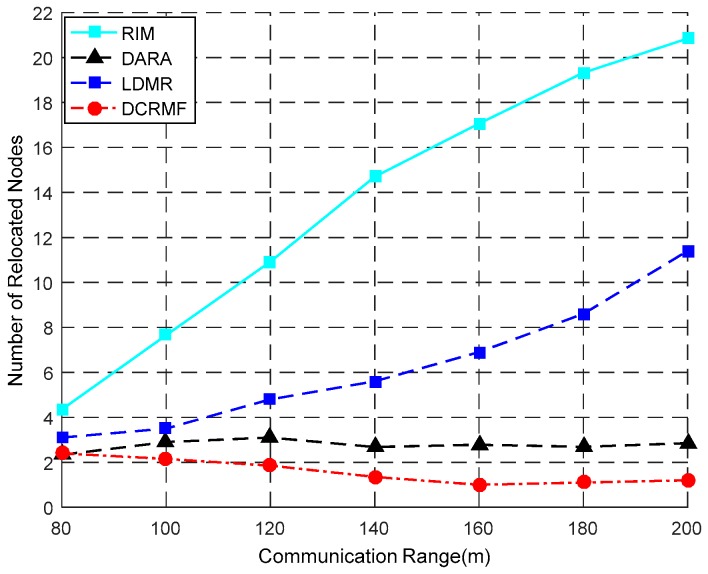
The relationship between the number of relocated nodes and the communication range by using the four algorithms.

The relationship between the number of relocated nodes and the communication range is shown in Figure 12, and some detailed contrast data is shown in Table 7. NRN denotes the number of the relocated nodes, and R denotes the communication range of the nodes. Obviously, RIM brings more computation complexity with the increasing of the number of relocated nodes caused by the increasing number of neighbor nodes in higher density networks. Even in lower density networks, any node abnormality easily causes the cascaded movement of the relocated nodes, and makes the relocation scale of the nodes become very large. Compared with DARA, RIM and LDMR, the proposed DCRMF saves more movement overhead.

Table 7 illustrates that the numbers of relocated nodes for the four algorithms all vary with the varying communication range and the number of relocated nodes in DCRMF is gradually less than those in the other three algorithms as the communication range becomes larger.

A comparison conclusion can be made from the above experiments. The RIM algorithm only involves the movements of one-hop neighbor nodes, and the maximum moving distance for one node does not exceed half of the communication radius of the node, so it has the advantage of maintaining the energy balance of the whole network. However, with increases of the total number of nodes and the communication radius, the total number of relocated nodes and the total movement overhead will be obviously increased. For the DARA algorithm, there is not a large number of nodes involved in the restoration process, and the total moving distance is not very large. However, because it does not distinguish between the dominant nodes and non-dominating nodes, the migration movement could easily cascade, and this brings a lot of unnecessary overhead to the network. The remarkable advantage of the LDMR algorithm is that it could achieve better performance on total moving distance. However, in the migration process, the LDMR algorithm involves every neighbor node around the abnormal node, so the total number of relocated nodes will be very large, and this could reduce the efficiency of the algorithm. Compared with the three algorithms above, the proposed DCRMF algorithm has better performances on the total number of relocated nodes and the total moving distance, which could effectively reduce the movement overhead of the nodes in the network.

## 6. Conclusions

WSANs are usually deployed in harsh environments where nodes’ abnormality has a high probability to happen. Such abnormities can lead to the situation in which the network connectivity becomes fragile or the network falls into a partitioned state. In this paper, a distributed autonomous connectivity restoration algorithm based on finite state machine is proposed. The criticality of the nodes is determined by using the connected dominating set, and the finite state machine mechanism is applied to confirm the critical nodes. Whether the connectivity restoration algorithm is initiated depends on the criticality of the abnormal nodes in the network topology. The proposed algorithm can effectively reduce the movement overheads of the nodes in the restoration process. Moreover, in order to adapt to the dynamic topology, the mechanism of updating the critical nodes regularly is introduced after performing the restoration algorithm. In the case of multiple node abnormities, a distributed regional restoration algorithm is introduced to restore the network connectivity. Experimental results indicate that, for different sizes and sparsity degrees of the network, particularly compared with the restoration algorithms without a distinction mechanism for critical nodes, the proposed algorithm not only has the better performance on less number of the total relocated nodes, but also has less total moving distances, and saves more movement overhead during the restoration process as well.

In some application scenarios, the network connectivity needs to be guaranteed as the number one priority, and next ensuring the large sensing coverage rate as far as possible is considered. This article mainly focuses on solving the critical problem of network connectivity restoration. Obviously, it could lead to a reduction of the network sensing coverage rate due to the appearance of some faulty nodes in the network, and it could also have an influence on the network sensing coverage when the node moves to other position. However, this influence will be limited to network systems with a certain number of redundant nodes, and also this influence will be limited for the application system with lower sensitive requirement to the sensing coverage rate. For some applications with higher sensitivity to network sensing coverage requirement, one needs to discuss the position optimization problem quantitatively for the nodes’ relocation, but this is not the critical issue discussed in this article.

## Figures and Tables

**Table 1:** Experimental parameter settings.

Parameter	Value
Minimum number of nodes	80
Maximum number of nodes	200
Number of node changing steps	20
Minimum communication range	80
Maximum communication range	200
Communication range changing step	20

**Table 2:** Comparison of the Reactive Algorithms.

Attribute	DARA	RIM	LDMR	DCRMF
Topology information	2-hop	1-hop	Muti-hop	2-hop
Max. distance by all nodes involved	(N − 3) ∗ *r*	(N − 1) ∗ *r*/2	(N_d_ − 1) ∗ *r*/2	(N_d_ − 1) ∗ *r*
Max. distance by each node	*r*	*r*/2	*r*/2	*r*
Segregate critical/non-critical	no	no	yes	yes
Max. relocation numbers	N − 3	N − 1	N_d_ − 1	N_d_ − 1

**Table 3:** Comparison of different methods on the change of total moving distance with the total number of nodes varying.

Algorithm	TMD (N = 80)	TMD (N = 100)	TMD (N = 120)	TMD (N = 140)	TMD (N = 160)	TMD (N = 180)	TMD (N = 200)
DCRMF	89.6	82.4	87.4	91.2	81.5	78.4	75.1
DARA	98.7	109.6	110.9	104.7	169.1	179.8	212.4
LDMR	172.3	165.4	143.2	124.1	118.7	103.5	92.3
RIM	154.3	197.6	254.3	348.8	362.7	451.7	572.8

**Table 4:** Comparison of different methods on the change of average number of relocated nodes with the numbers of nodes varying.

Algorithm	NRN (N = 80)	NRN (N = 100)	NRN (N = 120)	NRN (N = 140)	NRN (N = 160)	NRN (N = 180)	NRN (N = 200)
DCRMF	1.4	1.5	1.4	1.3	1.2	1.0	1.2
DARA	2.9	3.2	4.0	4.2	5.6	6.0	6.1
LDMR	6.2	7.9	9.7	10.8	12.4	12.9	13.4
RIM	5.6	7.1	9.1	11.9	13.3	16.2	20.4

**Table 5:** Comparison of different methods on the change of average number of total sending messages with the numbers of nodes varying.

Algorithm	TSM (N = 80)	TSM (N = 100)	TSM (N = 120)	TSM (N = 140)	TSM (N = 160)	TSM (N = 180)	TSM (N = 200)
DCRMF	7.1	6.3	5.7	5	4.7	3.5	3.2
DARA	5.2	5.6	7.4	8.5	9.1	11.9	14.2
LDMR	10.4	12.7	13.5	17.8	21.2	28.1	46.1
RIM	12.6	16.3	19.1	25.4	34.3	45.1	57.8

**Table 6:** Comparison of different methods on the change of total moving distance with the communication range varying.

Algorithm	TMD (R = 80)	TMD (R = 100)	TMD (R = 120)	TMD (R = 140)	TMD (R = 160)	TMD (R = 180)	TMD (R = 200)
DCRMF	87.6	94.7	100.2	108.7	112.4	118.6	121.0
DARA	89.4	108.4	162.7	174.5	189.2	211.9	294.3
LDMR	148.3	132.5	127.6	109.2	101.3	97.2	94.2
RIM	154.6	205.2	291.3	372.0	501.3	596.1	721.7

**Table 7:** Comparison of different methods on the change of number of relocated nodes with the communication range varying.

Algorithm	NRN (R = 80)	NRN (R = 100)	NRN (R = 120)	NRN (R = 140)	NRN (R = 160)	NRN (R = 180)	NRN (R = 200)
DCRMF	2.3	2.1	1.9	1.7	1.5	1.6	1.6
DARA	2.2	3.0	3.2	2.7	2.8	2.6	2.8
LDMR	3.1	3.5	4.8	5.6	6.9	8.6	11.4
RIM	4.2	7.6	10.8	14.4	17.1	19.2	20.8
